# Polygenic Hypercholesterolemia and Cardiovascular Disease Risk

**DOI:** 10.1007/s11886-019-1130-z

**Published:** 2019-04-22

**Authors:** Mahtab Sharifi, Marta Futema, Devaki Nair, Steve E. Humphries

**Affiliations:** 1 Centre for Cardiovascular Genetics, Institute of Cardiovascular Science, University College London, 5 University St, London, WC1E 6JF UK; 2 Department of Clinical Biochemistry, the Royal Free London NHS Foundation Trust, Pond Street, London, NW3 2QG UK

**Keywords:** Familial hypercholesterolemia, Polygenic hypercholesterolemia, *LDLR* gene, *APOB* gene, *PCSK9* gene

## Abstract

**Purpose of the Review:**

**I**dentification of loci and common single-nucleotide polymorphisms (SNPs) that have modest effects on plasma lipids have been used to confirm or refute the causal role of lipid traits in the development of coronary heart disease (CHD), and as tools to identify individuals with polygenic hypercholesterolemia.

**Recent Findings:**

Several groups have reported on the use of SNP scores in distinguishing individuals with a clinical diagnosis of familial hypercholesterolemia (FH) with a monogenic or polygenic etiology. We review evidence that those with monogenic FH have worse prognosis and discuss the possible mechanisms for this and their management.

**Summary:**

Individuals with a clinical phenotype of FH and a monogenic cause are at greater risk of CHD than those where no causative mutation can be found. The patients with polygenic hypercholesterolemia would not require elaborate cascade screening or secondary care input for their management.

## Introduction

The causal relationship between low-density lipoprotein cholesterol (LDL-C) and coronary atherosclerosis has been shown in several previous studies [1••]. Multiple randomized trials have demonstrated that lowering LDL-C by treatment with a statin reduces the risk of major coronary events [2].

In people with similar total cholesterol levels, different rates of coronary heart disease (CHD) events have been reported. It is well established that diet, physical inactivity, smoking, elevated blood pressure, diabetes, obesity, and thrombogenic factors play important roles in increasing risk of CHD. The contribution of these modifiable risk factors to developing a future coronary event is considerable, particularly in combination with non-modifiable personal characteristics such as age, male gender, and family history of early-onset CHD [3]. Results from a 12-year follow-up of 316,099 men screened for the multiple risk factor intervention trial (MRFIT) showed a strong graded relationship between serum cholesterol levels and CHD mortality [4].

Different factors are known to increase LDL-C concentration. The secondary causes such as diabetes mellitus, hypothyroidism, obesity, nephrotic syndrome, and excess alcohol intake are well-known. Among the hereditary causes, it is important to differentiate between monogenic and polygenic hypercholesterolemia as this might affect the patient’s management in long-term.

## Polygenic Hypercholesterolemia

Polygenic hypercholesterolemia is a common cause of elevated serum cholesterol concentrations. It represents the cases with a raised LDL-C with serum triglyceride concentrations within the reference range. Some patients with mixed dyslipidemias (elevations of both LDL-C and triglycerides) may also have polygenic hypercholesterolemia along with another condition such as metabolic syndrome or obesity.

Polygenic hypercholesterolemia is sometimes difficult to be differentiated clinically from familial hypercholesterolemia (FH). The presence of a mutation in any one of three common genes is responsible for causing FH. Most commonly, a mutation is found in the *LDLR* gene encoding the LDL receptor, or in the *APOB* gene encoding for apolipoprotein B, which is the major protein of the LDL particle, and a single mutation p.Arg3527Gln is found in about 5% of clinical FH patients in the UK. Finally, and least frequently, FH can be caused by a gain-of-function mutation in the *P*CSK9 gene (proprotein convertase subtilisin kexin 9), encoding the PCSK9 protein responsible for degradation of the LDL receptor during its intracellular recycling. FH is diagnosed either on phenotypic criteria, involving an elevated LDL-C level plus a family history of elevated LDL-C, premature CHD, or with a genetic testing [5]. However, a mutation in the above genes can only be identified in about 40–60% of people with clinically suspected FH [6], raising the question of what is the genetic etiology in the remainder.

Although some patients with no detected mutation may have a mutation in a yet-to-be identified gene, in the majority of this group, we now believe that there is a polygenic explanation. The Global Lipid Genetic Consortium (GLGC) meta-analysis of genome-wide association study identified 95 loci where common variants affect LDL-C level [7]. These loci contribute not only to normal variation in lipid traits but also to extreme lipid phenotypes. Talmud et al. showed that an accumulation of common small-effect LDL-C-raising alleles could increase the LDL-C level as high as the level in monogenic FH patients and cause polygenic hypercholesterolemia [6]. The 12 single-nucleotide polymorphisms (SNPs) used in this score are shown in Table 1. A “weighted” score improved accuracy, and was created by multiplying carriage of the LDL-C raising allele of the SNPs by the GLGC weight. Futema et al. found that addition of 21 LDL-C-raising SNPs did not si**g**nificantly improve the ability of the SNP score to discriminate between polygenic hypercholesterolemia and healthy subjects and a weighted score of six SNPs performed as well as the 12-SNP score. Using this approach, this study confirmed the lower SNP score in samples of patients with no-mutation from six European countries, demonstrating the robustness of the tool [9, 10].

**Table 1:** Global Lipid Genetic Consortium 12 SNP LDL-C gene score, showing the LDL-C-raising allele

CHR	SNP	Gene	Minor*	Common*	GLGC Weight for Score	MAF WHII
1	rs2479409	***PCSK9***	**G**	A	0.052	0.35
1	rs629301	*CELSR2*	G	**T**	0.15	0.21
2	rs1367117	***APOB***	**A**	G	0.10	0.33
2	rs4299376	*ABCG8*	**G**	T	0.071	0.32
6	rs1564348	*SLC22A1*	C	**T**	0.014	0.17
6	rs1800562	*HFE*	A	**G**	0.057	0.07
6	rs3757354	*MYLIP*	T	**C**	0.037	0.21
11	rs11220462	*ST3GAL4*	**A**	G	0.050	0.13
14	rs8017377	*KIAA1305*	**A**	G	0.029	0.48
19	rs6511720	***LDL-R***	T	**G**	0.18	0.13
19	rs429358	***APOE*** ^***ψ***^	C	T		0.15
19	rs7412	***APOE*** ^***ψ***^	T	C		0.08
19	ε2ε2	*APOE*			− 0.9	
19	ε2ε3	*APOE*			− 0.4	
19	ε2ε4	*APOE*			0.2	
19	ε3ε3	*APOE*			0	
19	ε3ε4	*APOE*			0.1	
19	ε4ε4	*APOE*			0.2	

^*^Risk alleles (LDL-C-raising) are indicated in **bold**. MAF, minor allele frequency. WHII is a UK-based study of healthy Caucasian subjects (*n* = 3020)

^ψ^APOE weights were based on haplotypic effects taken from [8]. Genes shown in **bold** are those where rare mutations of large effect on function cause FH

We estimated that in more than 80% of those with a clinical diagnosis of autosomal dominant FH but with no detectable mutation in *LDLR/APOB/PCSK9*, the polygenic explanation is most likely cause of their hypercholesterolemia [10]. In the remainder, a mutation in a novel gene may be present [6]. In an audit of the genetic testing that incorporated 12 LDL-C-raising SNPs associated with LDL-C concentrations above the diagnostic threshold for FH, it was found that approximately 36% of the cases had an FH-causing mutation and among the patients where no mutation was found, 54% had a SNP score consistent with an increased likelihood of a polygenic hypercholesterolemia [11].

Talmud et al. proposed that only those with a monogenic cause for their phenotype be given the diagnosis of FH, and the remainder be termed “Polygenic Hypercholesterolemia” [6]. In the polygenic group, cascade testing will be less cost-effective, since only about 30% of relatives will have elevated LDL-C compared to the 50% seen in monogenic families [12]. Clearly, those with a polygenic cause of their hypercholesterolemia will need and benefit from lipid lowering therapy, but the question remains as to whether their future CHD risk is as great as those with monogenic FH.

Factors that complicate clinical diagnostic accuracy of monogenic and polygenic hypercholesterolemia is the presence of multiple genes that have a small positive effect on LDL-C concentration, raising levels to those consistent with FH, or the presence of compensatory genes that lower LDL-C below thresholds for FH diagnosis. As an example of this, the Arg46Leu variant in *PCSK9* is a loss of function variant that is associated with ~ 12% lower LDL-C levels in subjects in the general population but with ~ 28% lower CHD risk [13]. This confirms the LDL-C burden hypothesis (see later), where lifelong low LDL-C is associated with a significant reduction in CHD risk.

## Atherosclerosis

The number of studies carried out to evaluate the severity of the atherosclerosis in polygenic hypercholesterolemia is very limited. Many papers report that the prevalence of CHD is higher in FH patients where a mutation was found compared to those with a clinical diagnosis of FH where no mutation can be found. Support for this also comes from the UK Simon Broome register where patients with a clinical diagnosis of definite FH had a 35% higher standardized mortality ratio (SMR) for CHD than those with a clinical diagnosis of possible FH (SMR 2.4 vs. 1.78) [14]. We know that up to 80% of those with clinical diagnosis of definite FH will carry an FH-causing mutation while by contrast, a mutation is detected in only 25–30% of possible FH patients. This means that at least 80% are likely to have a polygenic cause of their FH phenotype, and therefore the much lower death rate from CHD in the possible FH patients supports the view that the amount of coronary atherosclerosis in polygenic hypercholesterolemia will be less.

The higher CHD risk in FH subjects with a monogenic detected mutation has been recently shown in a study for the USA [15••]. Using next-generation sequencing (NGS) for the known FH genes among 20,485 CHD-free individuals, 1386 (6.7%) had LDL-C > 4.9 mmol/l (189 mg/L), and of these, 24 (1.7%) carried a known FH mutation. As expected, in the no-mutation group, increasing levels of LDL-C were associated with higher risk of CHD, with individuals with LDL-C > 4.9 mmol/l (189 mg/L) having a sixfold higher risk for CHD than those with LDL-C < 3.7 mmol/l (144 mg/dL). However, over the entire spectrum of LDL-C, those with an identified FH-causing mutation had 2–3-fold-elevated CHD risk compared to those with the same LDL-C but with no identified mutation (i.e., a polygenic plus environmental cause of their elevated lipid level).

Finally, we have recently demonstrated that in polygenic patients who had an LDL-C level as high as the monogenic FH, carotid intima media thickening (cIMT) and coronary calcification were lower than the monogenic FH patients [16•]. We compared the severity of carotid and coronary subclinical atherosclerosis between these two groups who had no previous evidence of cardiovascular disease. All individuals were treated with lipid-lowering therapy and no differences were seen in on-treatment lipid levels, as well as in other conventional atherosclerosis risk factors between groups. As shown in Table 2, after adjustment for age and gender, the means of cIMT measurements were significantly higher in several regions of the carotid tree in monogenic FH patients. In addition, coronary artery calcification (CAC) were significantly greater in 124 monogenic than 42 polygenic patients recruited form hospitals in the UK and Holland, with a greater prevalence of individuals with CAC > 100 (associated with elevated cardiovascular risk), observed in those with monogenic FH. Therefore, while CHD risk is high in polygenic hypercholesterolemia compared to the general population, the severity of the disease might not be the same as the monogenic FH.

**Table 2:** Characteristics and CIMT measure of patents with monogenic FH and polygenic hypercholesterolemia [16•]

		Monogenic *N* = 56	Polygenic *N* = 30	*P* value
Patients characteristics				
Male	*N* (%)	22 (40)	14 (47)	0.3
Age (years)	Mean (SD)	50 (14)	57 (12)	0.03
Total cholesterol (mmol/L)	Mean (SD)	8.1 (1.5)	8.2 (1.0)	0.5
LDL-cholesterol (mmol/L)	Mean (SD)	5.8 (1.6)	5.9 (0.9)	0.8
HDL-cholesterol (mmol/L)	Mean (SD)	1.5 (0.4)	1.9 (1.1)	0.1
Triglycerides (mmol/L)	Mean (SD)	1.2 (0.5)	1.6 (0.7)	0.01
On lipid-lowering drug	%	75	85	0.7
CIMT measures				
Mean cIMT (mm)	Mean (SD)	0.74 (0.70–0.79)	0.66 (0.61–0.72)	0.03
Mean bifurcation cIMT (mm)	Mean (SD)	0.81 (0.74–0.89)	0.70 (0.62–0.79)	0.05
Mean ICA cIMT (mm)	Mean (SD)	0.74 (0.66–0.83)	0.60 (0.52–0.7)	0.04

*CIMT* carotid intima media thickness, *ICA* internal carotid artery

## Why Is the Onset of CHD Earlier in Monogenic vs. Polygenic Hypercholesterolemia?

Premature CHD is an established phenomenon of FH, with the average age of onset of coronary symptoms shown to be significantly lower in men than women, with a mean age of 45 years compared to 55 years for women [17]. The risk and age of onset of atherosclerosis in people with monogenic FH tends to be proportional to the extent and duration of raised LDL-C, calculated as a cholesterol-year score [18]. This might be explained by the fact that the monogenic FH patients have had a raised cholesterol level since birth. The Simon Broome DNA study, where 410 definite FH patients were examined, showed that compared to those without an FH-causing mutation, in those with a mutation, the odds ratio for having CHD was 1.84 [19]. In patients with polygenic hypercholesterolemia, while the genetic susceptibility is still important, environmental factors such as obesity or metabolic disease might be additional contributing factors to the raised LDL-C level later in life.

**Fig. 1:**
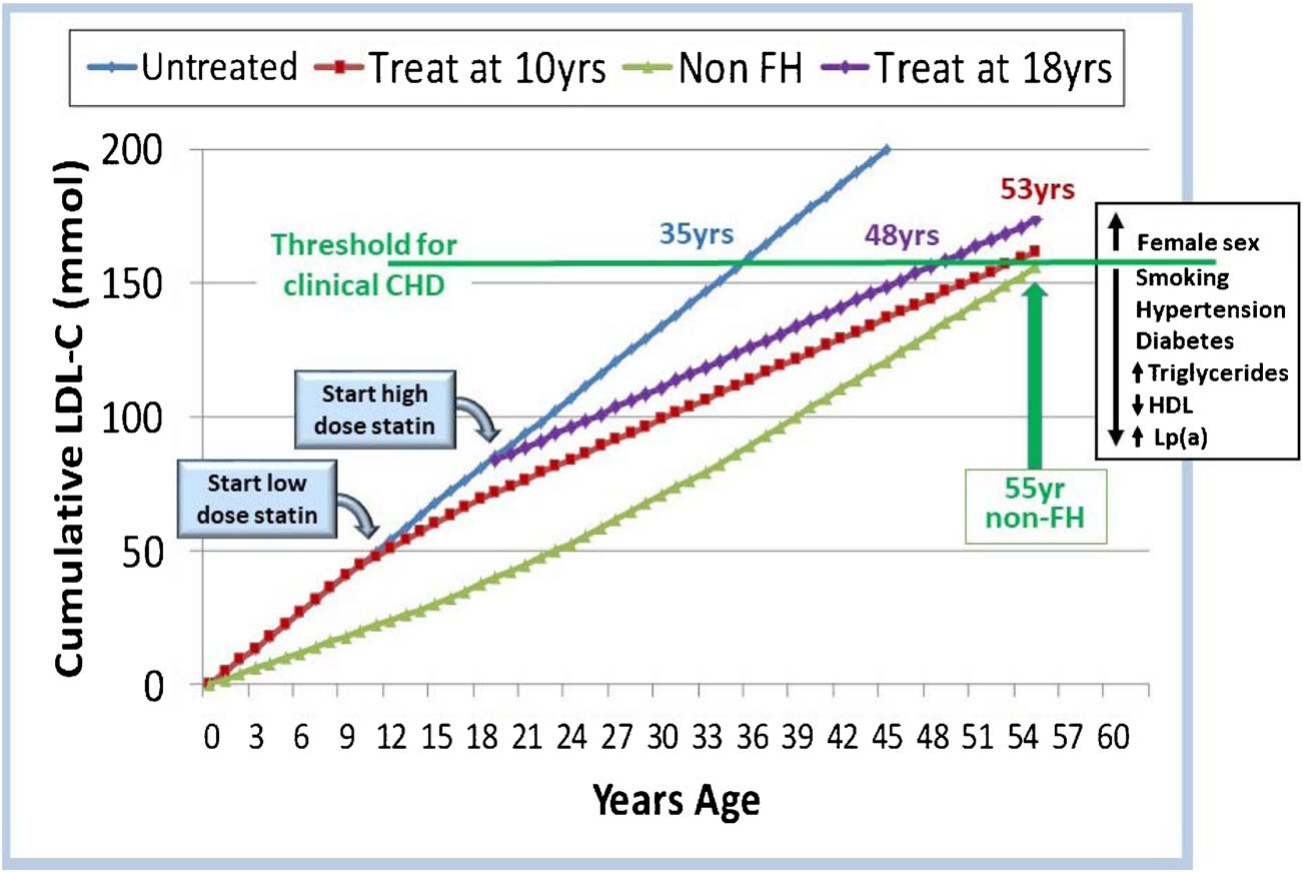
Cumulative LDL-burden, expressed as mmol/l per year, over a lifetime in non-FH and FH individuals with and without treatment showing threshold for CHD. (Data from [18])

The consequence of this elevated LDL-C since birth leads to the concept of estimating an individual’s “LDL-C burden,” as is shown in Fig. 1. LDL-C burden is calculated as the sum of an individual’s usual LDL-C multiplied by their age. In Fig. 1, the threshold for clinical CHD is set as the burden achieved by a non-FH subject at the age of 55 years. An untreated homozygous FH child reaches this level by the age of 12.5 years and a heterozygous FH adult by the age of 35 years. The cumulative LDL-C burden by the age of 18 years is 15% lower in FH patients treated with a low-dose statin from the age of 10 years onwards than in untreated FH patients, and the clinical threshold will be reached at 53 years. By contrast, delaying the start of any statin treatment until 18 years means that the threshold will be reached by 48 years, suggesting this delay could reduce healthy life expectancy by 8 years. Other personal characteristics that may influence age of crossing the potential clinical threshold such as gender, smoking history, etc. are shown in the box in Fig. 1. Confirming this view, a meta-analysis involving 312,00 participants showed that long-term exposure to lower LDL-C at early stages in life was associated with a threefold greater reduction in the risk of CHD for each unit of LDL-C than that observed during treatment with a statin started later in life. [1••]

## Management and Conclusions

The results of the UK Simon Broome Register have shown that since the introduction of statins, there has been a reduction in coronary mortality in FH patients by more than a third, and that in those with no CHD at registration, the CHD rate in treated FH patients is no greater than in the general population. Recent analysis of the Register data has confirmed the significant reduction in fatal CHD in men over the last 20 years, but with a smaller benefit seen in women, suggesting that women may not be being treated so often with high intensity statins [12]. The PCSK9 inhibitors represent a major new contribution to treatment of severe forms of FH.

Severe hypercholesterolemia needs to be treated independently of the genetic defect, due to the undisputable causal role LDL-C in atherosclerosis. Clearly, all patients with a clinical diagnosis of FH will need LDL-C levels reduced significantly, with UK guidelines suggesting by at least 50% from baseline (https://www.nice.org.uk/guidance/cg71), and European guidelines to below 2.5 mmol/l [20]. However, because of the higher LDL-C burden and in consequence higher atherosclerotic burden in the monogenic patients, this group needs particularly intensive LDL-C-lowering, which can best be achieved under the management of a lipid specialist. For some patients, to achieve LDL-C target values, may require treatment with multiple agents, and in some may include use of PCSK9 inhibitors. By contrast, in those who do not have a monogenic cause for their lipid phenotype, it is appropriate to estimate their CHD risk using risk algorithms such as QRISK2, and they could be managed in general practice [6]. The cascade testing recommended in the families of index cases with a monogenic cause is *not* recommended in those with polygenic hypercholesterolemia, because the number of “affected” relatives will be much less than the 50% found in those with a monogenic cause, and the process will be much less cost-effective.

The use of genetic information to stratify patients with a clinical diagnosis of FH into those with a monogenic or polygenic cause, and to have different management care-pathways, is a paradigm example of the utility of genetic in Precision Medicine. As NGS becomes cheaper, and the bioinformatics analysis has developed further, this may expand to whole genome sequencing to give an individual a more complete picture of their future risk of disease. It is also possible that the large inter-individual variability in the LDL-C response to a given dose of statins or PCSK9 inhibitors seen in different individuals may be caused to some extent by the genetic differences causing the FH phenotype in monogenic patients compared to those with polygenic hypercholesterolemia. If this could be demonstrated by future research, this may prove helpful in influencing monitoring and treatment strategies.

By generating a polygenic SNP score in diagnostic genetic laboratories, the likelihood of a polygenic etiology in mutation-negative patients with a clinical diagnosis of FH can be reported to assist their management. Mendelian randomization and genome-wide association studies have given significant insights into the role of genetics in dyslipidemia and cardiovascular risks. This has enabled the creation of genetic risk scores that have demonstrated improved risk prediction when added to clinical markers.
